# Machine Learning Prediction of Therapeutic Outcome After Transforaminal Epidural Steroid Injection for Radiculopathy from Herniated Lumbar Disc

**DOI:** 10.3390/bioengineering13010018

**Published:** 2025-12-25

**Authors:** Jeoung Kun Kim, Min Cheol Chang

**Affiliations:** 1 Department of Business Administration, School of Business, Yeungnam University, Gyeongsan-si 38541, Republic of Korea; kimjk70@yu.ac.kr; 2 Department of Physical Medicine & Rehabilitation, College of Medicine, Yeungnam University, Daegu 42415, Republic of Korea

**Keywords:** herniated lumbar disc, transforaminal epidural steroid injection, deep learning, machine learning, outcome prediction

## Abstract

Background/Objectives: Transforaminal epidural steroid injection (TFESI) is widely used to treat lumbosacral radicular pain caused by a herniated lumbar disc (HLD). However, therapeutic response varies substantially, and reliable outcome prediction remains challenging because of the multifactorial interplay of clinical and morphological factors. Machine learning (ML) approaches may address this limitation by modeling nonlinear interactions among patient-specific variables. Methods: This retrospective cohort study analyzed 242 patients with HLD-related radiculopathy who underwent single-level lumbar TFESI. Eight variables—age, sex, injection side, injection level, pain duration, pretreatment numeric rating scale (NRS) score, HLD location, and HLD subtype—were used as input features. Therapeutic outcome was defined as a ≥50% reduction in NRS score at 1 month after TFESI. Three predictive models, namely deep neural network (DNN), random forest (RF), and XGBoost, were developed and evaluated using a validation cohort of 49 patients. Results: The DNN model demonstrated the best validation performance, achieving an area under the curve (AUC) of 0.821 (95% confidence interval [CI], 0.690–0.929). The performance of the RF (AUC, 0.711; 95% CI, 0.535–0.865) and XGBoost (AUC, 0.674; 95% CI, 0.498–0.831) models was inferior to that of the DNN. In addition, the DNN produced fewer false-positive predictions and showed more robust discrimination between favorable and poor outcomes than the other ML models. Conclusions: A deep learning–based predictive model demonstrated superior performance in predicting therapeutic outcomes after lumbar TFESI in patients with HLD-related radiculopathy. Integration of routine clinical and magnetic resonance imaging (MRI)-derived features into ML algorithms may enhance individualized prognostication and assist clinicians in optimizing patient selection for interventional procedures. To the best of our knowledge, this is the first study to develop an ML-based model integrating routine clinical variables with MRI findings for the prediction of TFESI outcomes in HLD-related radiculopathy. Nevertheless, the study is limited by its single-center retrospective design, lack of external validation, and reliance on MRI assessments performed by a single rater. Future multicenter studies are warranted to improve generalizability and confirm clinical utility.

## 1. Introduction

A herniated lumbar disc (HLD) is a common cause of lumbosacral radicular pain and results from both mechanical compression of the nerve root and biochemical irritation mediated by inflammatory factors [1,2]. Radiculopathy secondary to HLD is frequently associated with severe radiating leg pain, functional limitation, and reduced quality of life [3,4]. Although many patients respond to conservative management, interventional procedures may be required when symptoms persist or progress despite adequate medical therapy [5].

Transforaminal epidural steroid injection (TFESI) is widely used for the management of HLD-related radicular pain [5,6]. The technique delivers corticosteroids and local anesthetics adjacent to the affected nerve root, thereby attenuating inflammatory activity and reducing chemical irritation [7,8]. However, clinical response varies substantially among patients. Several clinical and imaging variables—including age, duration of symptoms, herniation level, and herniation subtype—have been investigated as potential predictors of treatment outcome, although their individual predictive value remains limited [9,10,11,12].

Traditional statistical methods have been used to examine associations between magnetic resonance imaging (MRI) findings or clinical parameters and TFESI outcomes; however, these approaches are limited in their capacity to accommodate multiple interacting variables and nonlinear relationships [9,10,11,12]. Therapeutic response in HLD likely reflects a complex interplay of demographic, clinical, and morphological factors, making outcome prediction using conventional methods challenging. In particular, traditional regression models are based on linear and additive assumptions and therefore cannot adequately represent the complex, nonlinear interactions between clinical variables and MRI-derived morphological characteristics associated with TFESI outcomes. Consequently, important multidimensional patterns may remain undetected, contributing to the inconsistent predictive results reported in previous studies [9,10,11,12]. In contrast, nonlinear machine learning (ML) algorithms—particularly deep neural networks (DNNs)—provide greater flexibility to model high-dimensional, nonlinear relationships and variable interactions, thereby more effectively capturing the complexity of real-world clinical scenarios.

Recent advances in ML and deep learning (DL) have enabled promising approaches for clinical outcome prediction [13,14,15]. ML algorithms can analyze structured clinical data and identify latent patterns without explicit programming, whereas DL algorithms—particularly neural networks—are capable of modeling high-dimensional, nonlinear relationships within complex datasets [16,17]. These techniques have demonstrated strong predictive performance across multiple domains of musculoskeletal and spine medicine, frequently outperforming traditional analytical methods [16,17].

Despite the growing application of ML and DL in pain and spine research, few studies have specifically applied these methods to predict TFESI outcomes in patients with HLD-related radiculopathy. To date, only one study by Kim et al. developed a DNN model trained exclusively on magnetic resonance images to predict TFESI outcomes in patients with HLD [18]. However, that study did not incorporate clinical variables and included a heterogeneous cohort comprising both HLD and lumbar spinal stenosis, limiting its applicability to HLD-related radicular pain. To our knowledge, no prior studies have developed DL-based predictive models for TFESI outcomes in patients with HLD that integrate both detailed clinical parameters and MRI-based morphological characteristics of disc herniation. Accurate prediction of TFESI response may enable clinicians to optimize decision-making, avoid unnecessary procedures, and provide more personalized prognostic information.

Accordingly, this study aimed to develop and validate ML-based predictive models—particularly a DNN—to classify therapeutic outcomes following lumbar TFESI in patients with HLD-related radiculopathy. Eight clinically relevant variables, encompassing demographic characteristics, symptom profiles, and MRI-defined herniation features, were used as input features. Model performance was compared with that of conventional ML algorithms to assess the relative predictive value of DL for predicting TFESI effectiveness in patients with lumbosacral radicular pain due to HLD.

## 2. Materials and Methods

### 2.1. Participants

This retrospective study included 242 patients who visited the spine center of a university hospital and underwent lumbar TFESI for the treatment of radicular pain caused by HLD between January 2013 and December 2021. Patient characteristics were as follows: mean age, 56.6 ± 14.8 years; sex (men/women), 119:123; symptom laterality (right/left/bilateral), 106:114:22; injection levels (L2:L3:L4:L5:S1), 2:8:29:139:64; pain duration, 15.3 ± 32.8 months; pretreatment numeric rating scale (NRS; 0 = no pain, 10 = worst pain), 5.2 ± 1.5; HLD location (central/right or left central/foraminal or extraforaminal), 89:128:25; and HLD subtype (protrusion/extrusion/sequestration), 109:129:4. The inclusion criteria were as following: (1) single-level lumbar TFESI for segmental lower-extremity radiating pain attributable to HLD; (2) ≥50% temporary pain relief following a diagnostic nerve root block with 1 mL of 2% lidocaine; and (3) MRI and electrophysiological findings concordant with the clinical presentation. The study protocol was approved by the Institutional Review Board of Yeungnam University Hospital, which waived the requirement for written informed consent because of the retrospective study design.

### 2.2. TFESI Procedures

TFESI was performed using a standardized technique as previously described [19]. All procedures were carried out by a single interventional physiatrist with expertise in spinal injections under strict aseptic conditions. Patients were positioned prone, and C-arm fluoroscopy (Siemens, Erlangen, Germany) was used to identify the target level and guide needle placement. The skin entry site was anesthetized with 1% lidocaine. A 25-gauge, 90 mm spinal needle with a bent tip was advanced to a position between the lateral aspect of the vertebral body and the 6 o’clock position beneath the pedicle. Lateral fluoroscopic imaging confirmed that the needle tip was located between the spinal laminar margin and the posterior vertebral body. Under anteroposterior fluoroscopy, 0.3 mL of nonionic contrast medium was injected to confirm the absence of intravascular uptake and appropriate epidural spread within the neural foramen. Contrast injection was repeated under real-time fluoroscopic monitoring, followed by administration of 20 mg (0.5 mL) of triamcinolone, 0.5 mL of bupivacaine hydrochloride, and 1 mL of normal saline.

### 2.3. Input Data for the DL Algorithm

Eight variables were used as input features for the DL model: age, sex, side, injection level, pain duration, pretreatment NRS, HLD location, and HLD subtype. Although additional MRI metrics—such as canal diameter, disc height, herniated disc size, Modic changes, and Pfirrmann grade—were available, only these eight predictors were selected because they represent core variables routinely collected in clinical practice. HLD location and subtype were evaluated by a single physiatrist with more than 15 years of experience in musculoskeletal disorders. Disc location was determined on axial lumbar MRI and categorized as central, right or left central, foraminal, or extraforaminal. Disc subtype was assessed on axial and sagittal lumbar MRI and classified as protrusion, extrusion, or sequestration according to morphological characteristics, in accordance with the North American Spine Society nomenclature and classification of lumbar disc pathology [20].

### 2.4. Output Data for the DL Algorithm (Therapeutic Outcome)

Pain severity was assessed at baseline and at the 1-month follow-up after lumbar TFESI using the NRS. NRS data were obtained through chart review. A favorable outcome was defined as a ≥50% reduction in the NRS score at 1 month compared with the pretreatment value, whereas a poor outcome was defined as a <50% reduction [16,21]. The percentage change in NRS score was calculated to quantify changes in pain severity:

$$Change\;in\;NRS\left(\%\right)=\frac{Pretreatment\;NRS\;score-1-month\;post-TFESI\;NRS\;score}{Pretreatment\;NRS\;score\;}\times 100$$

Among the study population, 147 patients experienced a favorable outcome, while 95 patients exhibited a poor outcome.

### 2.5. Data Processing and Model Development

The dataset comprising 242 patients was preprocessed and randomly partitioned into training (n = 193; 79.8%) and validation (n = 49; 20.2%) sets. The binary therapeutic outcome served as the classification target. Eight input variables were included: three numerical features (age, pain duration, and pretreatment NRS score) and five categorical features (sex, injection side, injection level, HLD location, and HLD subtype). The therapeutic outcome was treated as a categorical variable.

Three ML models—a DNN, random forest (RF) [22], and extreme gradient boosting (XGBoost) classifier [23]—were developed to predict TFESI outcomes. Model-specific preprocessing was applied: for the DNN, numerical features were standardized to zero mean and unit variance, and categorical features were one-hot encoded; for XGBoost, categorical features were label encoded and processed using the algorithm’s native handling. To address class imbalance, the scale_pos_weight parameter, defined as the ratio of majority to minority class samples, was applied during XGBoost training. The final model architectures and optimized hyperparameters are summarized in Table 1.

## 2.6. Statistical Analysis

Model performance was evaluated using the area under the curve (AUC) from receiver operating characteristic (ROC) analysis. Ninety-five percent confidence intervals (CIs) for the AUC were calculated using a 10,000-iteration bootstrapping method. All analyses were performed in Python 3.10.17, using scikit-learn 1.4.2 for statistical metrics and TensorFlow 2.17.1 for DL model development.

## 3. Results

**Figure 1:**
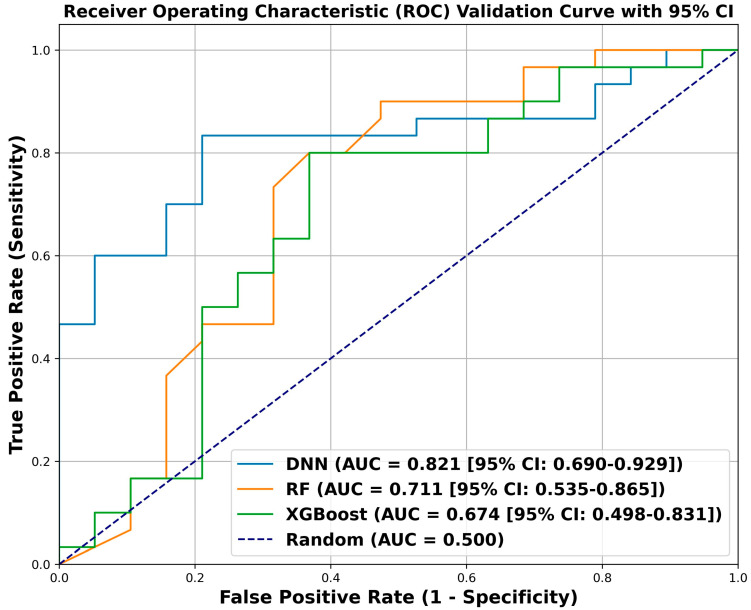
Receiver operating characteristic curves for the three models on the validation dataset. DNN, deep neural network; RF, random forest; AUC, area under the curve; CI, confidence interval.

**Figure 2:**
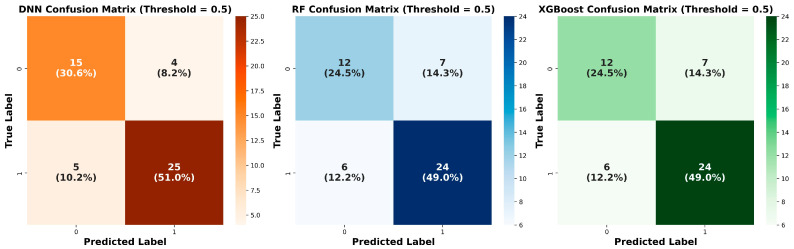
Confusion matrices for the DNN, RF, and XGBoost models. Label 0, poor outcome; Label 1, favorable outcome.

The developed DNN model consisted of three fully connected layers (128-64-32 neurons) with Sigmoid Linear Unit (SiLU) activation functions. It employed an AdamW optimizer with a learning rate of 9.551 × 10^−3^, weight decay of 1.0 × 10^−3^, batch size of 16, and no dropout regularization. The model achieved the highest validation accuracy of 0.816 (training accuracy, 0.881) and the highest validation AUC of 0.821 (95% CI: 0.690–0.929, along with a Brier score of 0.185, indicating good model calibration [24]), outperforming both RF and XGBoost (Figure 1). Classification metrics indicated balanced discrimination between poor and favorable outcomes. Precision values were 0.750 for poor outcomes and 0.862 for favorable outcomes, with corresponding recall values of 0.789 and 0.883, yielding F1-scores of 0.769 and 0.847, respectively (Table 2). The weighted average F1-score was 0.817, demonstrating robust overall classification performance. On the validation set, the DNN produced four false-negative and five false-positive predictions (Figure 2).

The RF model was configured with the following parameters: n_estimators = 10, max_depth = none, min_samples_split = 3, min_samples_leaf = 1, max_features = sqrt, bootstrap = true, class_weight = {0:1, 1:0.6462585034013606}, criterion = gini, and max_samples = none. The model achieved a validation accuracy of 0.735 (training accuracy, 0.933) and a validation AUC of 0.711 (95% CI, 0.536–0.867; Figure 1). Class imbalance (support: poor outcome = 19, favorable outcome = 30) was addressed using the class_weight parameter, with a weight of 1 assigned to the minority poor-outcome class and 0.646 to the majority favorable-outcome class, thereby increasing the penalty for misclassification of the minority class. Precision values were 0.619 for poor outcomes and 0.786 for favorable outcomes, with corresponding recall values of 0.684 and 0.733, yielding F1-scores of 0.650 and 0.759, respectively. The weighted average F1-score was 0.717. On the validation set, the RF model generated seven false-negative and six false-positive predictions (Figure 2).

The XGBoost model was configured with 60 estimators, a maximum tree depth of 4, and a learning rate of 0.05. Additional hyperparameters included subsample = 0.7, colsample_bytree = 1.0, gamma = 0.1, reg_alpha = 0.1, reg_lambda = 2.0, and scale_pos_weight = 1. The model achieved a validation accuracy of 0.735 (training accuracy, 0.845) and a validation AUC of 0.674 (95% CI, 0.500–0.833; Figure 1). Precision values were 0.667 for poor outcomes and 0.774 for favorable outcomes, with corresponding recall values of 0.632 and 0.800, yielding F1-scores of 0.649 and 0.787, respectively. The weighted average F1-score was 0.735. On the validation set, the XGBoost model produced seven false-negative and six false-positive predictions (Figure 2).

**Figure 3:**
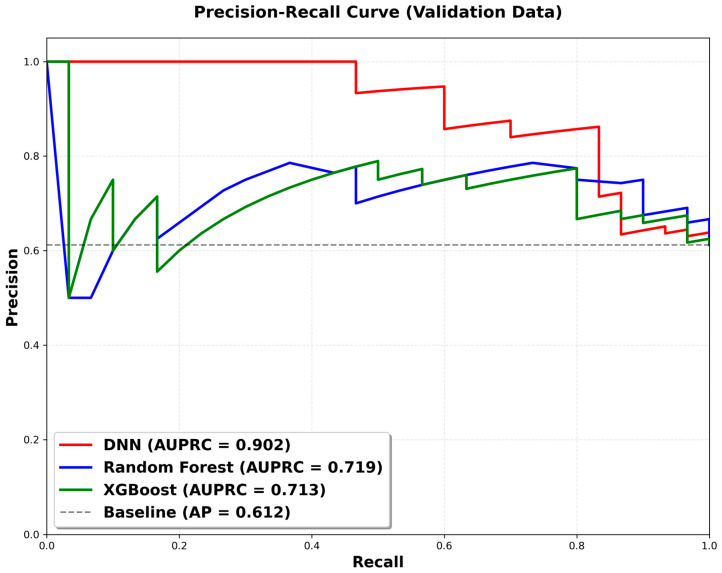
Precision–recall curves for the DNN, RF, and XGBoost models.

Figure 3 shows the precision–recall curves for the DNN, RF, and XGBoost models. The DNN demonstrated superior diagnostic performance, with an area under the precision–recall curve (AUPRC) of 0.902, markedly outperforming RF (0.719) and XGBoost (0.713). Notably, the DNN maintained perfect precision (1.0) up to a 45% recall threshold, ensuring zero false positives in high-confidence predictions—an important attribute for minimizing clinical alarm fatigue. These results highlight the robustness of the DL architecture in capturing complex nonlinear interactions, enabling precise patient stratification and offering a reliable alternative to conventional ML methods.

**Figure 4:**
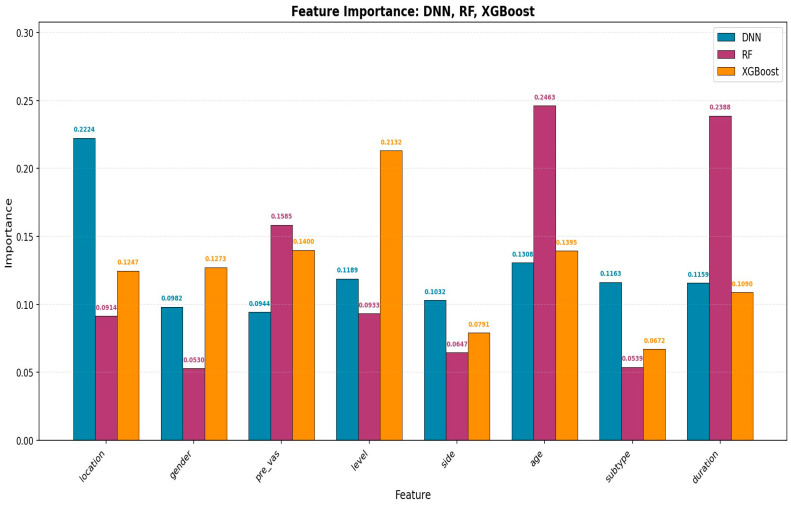
Comparison of feature importance across the DNN, RF, and XGBoost models.

Figure 4 illustrates the feature importance for the DNN, RF, and XGBoost models. For the RF and XGBoost models, intrinsic feature importance metrics were used. For the DNN model, SHapley Additive exPlanations (SHAP) analysis was applied to derive feature importance scores.

## 4. Discussion

In this study, we developed ML-based predictive models to classify therapeutic outcomes after lumbar TFESI in patients with radiculopathy caused by HLD. Among the evaluated models, the DNN achieved a validation AUC of 0.821 and a validation accuracy of 0.816, showing superior performance compared with RF and XGBoost. These findings suggest that nonlinear DL architectures may better capture the complex interactions among demographic, clinical, and morphological factors that influence TFESI response in patients with lumbar radiculopathy [25].

Previous studies examining predictors of TFESI outcomes have largely relied on traditional statistical methods, which have yielded inconsistent findings because of the multifactorial nature of radicular pain [9,10,11,12]. Variables such as symptom duration, baseline pain severity, and disc morphology have been proposed as potential predictors; however, their individual predictive value remains limited [9,10,11,12]. The present findings support accumulating evidence that heterogeneous clinical parameters can be integrated more effectively using ML approaches than with conventional analytical methods [25,26]. The superior performance of the DNN model likely reflects its ability to capture nonlinear data structures and uncover latent relationships that are not readily identified by linear or tree-based models [27,28].

The relatively high precision in classifying favorable outcomes and strong recall for identifying responders are clinically meaningful. Accurate prediction of TFESI success may enable clinicians to set realistic expectations, optimize patient selection, and reduce unnecessary procedures. Notably, the DNN produced fewer false-positive predictions than the other models, which is particularly relevant in interventional settings, where overestimation of benefit could lead to unwarranted injections. Collectively, these findings support the potential role of DL-based prediction tools as adjuncts to clinical decision-making in spine care.

This study has several limitations. First, MRI findings were evaluated by a single physiatrist, which may introduce observer bias and affect the reliability of input data and model performance. Second, external validation was not possible due to the single-center design; multi-institutional datasets are needed to confirm generalizability across populations with varying clinical and imaging characteristics. Third, therapeutic outcomes were assessed only at 1 month post-TFESI, reflecting short-term steroid effects rather than long-term outcomes. The absence of 3- to 6-month follow-up data represents an important limitation. Fourth, a comprehensive cost-of-error matrix was not established. Finally, model development was limited to DNN, RF, and XGBoost, and other ML algorithms were not explored. Despite these limitations, this study demonstrates the feasibility and clinical relevance of using ML models to predict TFESI outcomes in patients with HLD-related radiculopathy. ML algorithms, particularly DL models, could serve as valuable adjuncts to individualized treatment planning by integrating routinely collected clinical and imaging data.

## 5. Conclusions

The DNN model demonstrated superior predictive performance, outperforming both RF and XGBoost, and effectively captured complex interactions among demographic, clinical, and MRI-derived features. To the best of our knowledge, this is the first study to develop an ML-based model that integrates routine clinical variables with MRI findings to predict TFESI outcomes in patients with HLD-related radiculopathy. These findings underscore the potential of ML—particularly DL—to support individualized treatment planning, improve prognostic accuracy, and optimize patient selection for interventional procedures.

## Figures and Tables

**Table 1:** Model structures of the DNN, RF, and XGBoost models.

ML Model	Model Structure
DNN	- Three layers with 128-64-32 neurons, learning rate 0.009551, AdamW optimizer, SiLU activation, batch size 16, weight decay 0.001, dropout 0 - Training accuracy 0.881, validation accuracy 0.816 - Validation AUC 0.821 with 95% CI (0.690–0.929)
RF	- n_estimators 10, max_depth None, min_samples_split 3, min_samples_leaf 1, max_features sqrt, bootstrap True, class_weight {0:1, 1:0.6462585034013606}, criterion: gini, max_samples None - Training accuracy 0.933, validation accuracy 0.735 - Validation AUC 0.711 with 95% CI (0.536–0.867)
XGBoost	- n_estimators 60, max_depth 4, learning_rate 0.05, subsample 0.7, colsample_bytree 1, gamma 0.1, reg_alpha 0.1, reg_lambda 2, scale_pos_weight 1, - Training accuracy 0.845, validation accuracy 0.735 - Validation AUC 0.674 with 95% CI (0.500–0.833)

Abbreviations: ML, machine learning; DNN, deep neural network; RF, random forest; XGBoost, extreme gradient boosting; AdamW, adaptive moment estimation; AUC, area under the curve; CI, confidence interval.

**Table 2:** Model performance of the DNN, RF, and XGBoost models.

ML Model	Model Performance				
DNN	Class	Precision	Recall	F1-score	Support
	0 (poor outcome)	0.75	0.789	0.769	19
	1 (favorable outcome)	0.862	0.833	0.847	30
	Macro average	0.806	0.811	0.808	49
	Weighted average	0.819	0.816	0.817	49
RF	Class	Precision	Recall	F1-score	Support
	0 (poor outcome)	0.619	0.684	0.65	19
	1 (favorable outcome)	0.786	0.733	0.759	30
	Macro average	0.702	0.709	0.704	49
	Weighted average	0.721	0.714	0.717	49
XGBoost	Class	Precision	Recall	F1-score	Support
	0 (poor outcome)	0.667	0.632	0.649	19
	1 (favorable outcome)	0.774	0.8	0.787	30
	Macro average	0.72	0.716	0.718	49
	Weighted average	0.732	0.735	0.733	49

Class 0, poor outcome; Class 1, favorable outcome. Abbreviations: ML, machine learning; DNN, deep neural network; RF, random forest; XGBoost, extreme gradient boosting.

## Data Availability

All data are contained within the article.

## Abbreviations

The following abbreviations are used in this manuscript:

AUC	Area under the curve
CI	Confidence interval
CNN	Convolutional neural network
DL	Deep learning
DNN	Deep neural network
HLD	Herniated lumbar disc
ML	Machine learning
MRI	Magnetic resonance imaging
NRS	Numeric rating scale
RF	Random forest
ROC	Receiver operating characteristic
TFESI	Transforaminal epidural steroid injection

## References

[1] Kwak S.G., Choo Y.J., Kwak S., Chang M.C. (2023). Effectiveness of transforaminal, interlaminar, and caudal epidural injections in lumbosacral disc herniation: A systematic review and network meta-analysis. Pain Physician.

[2] Lee M.Y., Boudier-Revéret M., Cho H.K., Chang M.C. (2020). The successful treatment of herniated lumbar discs that are refractory to repeated epidural steroid injection by using a navigable percutaneous disc decompression device: A case series. J. Pain Res..

[3] Dydyk A.M., Khan M.Z., Singh P. (2025). Radicular Back Pain. StatPearls.

[4] Mahmutović E., Safiye T., Biševac E., Ajdinović A., Salihagić Z., Minić S., Dolićanin Z. (2024). Quality of life and functional status of patients with lumbar radiculopathy. Iran. J. Public Health.

[5] Agarawal S., Ramachandraiah M.K. (2023). Traditional Safe Triangle Approach Versus Kambin’s Triangle Approach: Does Approach Really Matter in Transforaminal Epidural Steroid Injection (TFESI) for Lumbar Disc Herniation?. Cureus.

[6] Liawrungrueang W., Cho S.T., Homlakhorn J., Sarasombath P. (2025). Comparative effectiveness of transforaminal epidural steroid injection: Subpedicular versus Kambin’s triangle technique: A single-centre experience. J. Spine Surg..

[7] Lee J.H. (2024). The Understanding and Appropriate Use of Corticosteroid in Epidural Injection: A Narrative Review. Int. J. Pain.

[8] Viswanathan V.K., Kanna R.M., Farhadi H.F. (2020). Role of transforaminal epidural injections or selective nerve root blocks in the management of lumbar radicular syndrome—A narrative, evidence-based review. J. Clin. Orthop. Trauma.

[9] Ekedahl H., Jönsson B., Annertz M., Frobell R.B. (2017). The 1-year results of lumbar transforaminal epidural steroid injection in patients with chronic unilateral radicular pain: The relation to MRI findings and clinical features. Am. J. Phys. Med. Rehabil..

[10] Park D.Y., Kang S., Park J.H. (2019). Factors predicting favorable short-term response to transforaminal epidural steroid injections for lumbosacral radiculopathy. Medicina.

[11] Sariyildiz M.A., Batmaz I., Hattapoğlu S. (2024). Predictors of successful treatment after transforaminal epidural steroid injections in patients with lumbar disc herniation. J. Back Musculoskelet. Rehabil..

[12] Wang M., Ling H., Zheng B., Song L. (2023). Predictors of a favorable response to transforaminal epidural steroid injections for lumbar radiculopathy in the elderly. Pain Physician.

[13] Rahman A., Debnath T., Kundu D., Khan M.S.I., Aishi A.A., Sazzad S., Sayduzzaman M., Band S.S. (2024). Machine learning and deep learning-based approach in smart healthcare: Recent advances, applications, challenges and opportunities. AIMS Public Health.

[14] Sadr H., Nazari M., Khodaverdian Z., Farzan R., Yousefzadeh-Chabok S., Ashoobi M.T., Hemmati H., Hendi A., Ashraf A., Pedram M.M. (2025). Unveiling the potential of artificial intelligence in revolutionizing disease diagnosis and prediction: A comprehensive review of machine learning and deep learning approaches. Eur. J. Med. Res..

[15] Sharma A., Lysenko A., Jia S., Boroevich K.A., Tsunoda T. (2024). Advances in AI and machine learning for predictive medicine. J. Hum. Genet..

[16] Kim J.K., Chang M.C. (2024). Convolutional neural network algorithm trained on lumbar spine radiographs to predict outcomes of transforaminal epidural steroid injection for lumbosacral radicular pain from spinal stenosis. Sci. Rep..

[17] Wang M.X., Kim J.K., Kim C.R., Chang M.C. (2024). Deep learning algorithm trained on oblique cervical radiographs to predict outcomes of transforaminal epidural steroid injection for pain from cervical foraminal stenosis. Pain Ther..

[18] Kim J.K., Wang M.X., Chang M.C. (2022). Deep learning algorithm trained on lumbar magnetic resonance imaging to predict outcomes of transforaminal epidural steroid injection for chronic lumbosacral radicular pain. Pain Physician.

[19] Kwak S., Jang S.H., Chang M.C. (2021). Long-term outcomes of transforaminal epidural steroid injection in patients with lumbosacral radicular pain according to the location, type, and size of herniated lumbar disc. Pain Pract..

[20] Fardon D.F., Milette P.C. (2001). Nomenclature and classification of lumbar disc pathology. Recommendations of the Combined task Forces of the North American Spine Society, American Society of Spine Radiology, and American Society of Neuroradiology. Spine.

[21] Ghahreman A., Ferch R., Bogduk N. (2010). The efficacy of transforaminal injection of steroids for the treatment of lumbar radicular pain. Pain Med..

[22] Greenberg M., Frid V. (2025). Physics-guided random forest classification of marine sediments using frequency-dependent acoustic reflection spectra. Appl. Sci..

[23] Chen T., Guestrin C. XGBoost: A scalable tree boosting system. Proceedings of the 22nd ACM SIGKDD International Conference on Knowledge Discovery and Data Mining.

[24] Brier G.W. (1950). Verification of forecasts expressed in terms of probability. Mon. Weather Rev..

[25] Topol E.J. (2019). High-performance medicine: The convergence of human and artificial intelligence. Nat. Med..

[26] Hanna M.G., Pantanowitz L., Dash R., Harrison J.H., Deebajah M., Pantanowitz J., Rashidi H.H. (2025). Future of Artificial Intelligence-Machine Learning Trends in Pathology and Medicine. Mod. Pathol. Off. J. United States Can. Acad. Pathol. Inc..

[27] Acici K. (2025). Comparative Analysis of Machine and Deep Learning Algorithms for Bragg Peak Estimation in Polymeric Materials for Tissue-Sparing Radiotherapy. Polymers.

[28] Esteva A., Robicquet A., Ramsundar B., Kuleshov V., DePristo M., Chou K., Cui C., Corrado G., Thrun S., Dean J. (2019). A guide to deep learning in healthcare. Nat. Med..

